# Exit from Synchrony in Joint Improvised Motion

**DOI:** 10.1371/journal.pone.0160747

**Published:** 2016-10-06

**Authors:** Assi Dahan, Lior Noy, Yuval Hart, Avi Mayo, Uri Alon

**Affiliations:** 1 Department of Molecular Cell Biology, Weizmann Institute of Science, Rehovot, Israel; 2 Theatre Lab, Weizmann Institute of Science, Rehovot, Israel; University of Münster, GERMANY

## Abstract

Motion synchrony correlates with effective and well-rated human interaction. However, people do not remain locked in synchrony; Instead, they repeatedly enter and exit synchrony. In many important interactions, such as therapy, marriage and parent-infant communication, it is the ability to exit and then re-enter synchrony that is thought to build strong relationship. The phenomenon of entry into zero-phase synchrony is well-studied experimentally and in terms of mathematical modeling. In contrast, exit-from-synchrony is under-studied. Here, we focus on human motion coordination, and examine the exit-from-synchrony phenomenon using experimental data from the mirror game paradigm, in which people perform joint improvised motion, and from human tracking of computer-generated stimuli. We present a mathematical mechanism that captures aspects of exit-from-synchrony in human motion. The mechanism adds a random motion component when the accumulated velocity error between the players is small. We introduce this mechanism to several models for human coordinated motion, including the widely studied HKB model, and the predictor-corrector model of Noy, Dekel and Alon. In all models, the new mechanism produces realistic simulated behavior when compared to experimental data from the mirror game and from tracking of computer generated stimuli, including repeated entry and exit from zero-phase synchrony that generates a complexity of motion similar to that of human players. We hope that these results can inform future research on exit-from-synchrony, to better understand the dynamics of coordinated action of people and to enhance human-computer and human-robot interaction.

## Introduction

When people interact productively they often synchronize their motion [1–9]. This synchrony has been studied in the fields of developmental psychology [10,11], social neuroscience [12–14], robotics [15,16], joint action [17–19] and coordination dynamics [20–24].

Studies in coordination dynamics showed that people tapping fingers [5,23] or rocking chairs [24] tend to synchronize and reach a shared rhythm [5,25]. They can synchronize either in phase or out-of-phase, but synchrony in-phase (zero-phase synchrony) is more stable especially at high frequencies. This behavior is captured by well-validated mathematical models, including the Haken, Kelso and Bunz model (HKB) [26–30]. Synchrony is also found in more complex settings [31–35] such as mother-infant interactions [36–38], conversations [39–43] and therapy [44,45]. Synchrony has been shown to correlate with good interpersonal outcomes such as enhanced cooperation [46,47] and rapport [4,17,48–50].

People, however, do not remain in synchrony continually even in the best interaction. Instead, people tend to go in and out of synchrony. For example, Tronick and co-workers showed that mother-infant interactions are synchronized well for only a small fraction of the time [51–53], and there are many ruptures of synchrony. Repair of such ruptures is thought to be an important aspect of infant development, as well as in strengthening interpersonal bonds in marriage and therapy [44,54]. Moreover, exit from established interactions open the possibility for expansion or discovery [11].

In terms of mathematical modelling, the attainment of zero-phase synchrony is well understood [4,5,20,26,55]. At heart, it relates to the propensity of coupled oscillator equations to synchronize [56]. However, the phenomenon of exit from zero-phase synchrony is under-studied [30]. It may be related to phenomena of meta-stability in the relative phase dynamics of coupled oscillators with different intrinsic frequencies [57]. Exit-from-synchrony is important in order to simulate and understand realistic human interaction: without it, people can become locked in repeating behavior which can be boring [58], and lack the possibility to explore new behavior and to repair ruptures which strengthen relationship. Therefore, there is a need for quantitative studies in order to experimentally characterize and mathematically model exit-from-synchrony.

To address the exit-from-synchrony phenomenon, we employ a recently introduced paradigm for joint improvised motion called the mirror game [59–64]. In the mirror game, two players move handles along parallel tracks, and are asked to create synchronized and interesting motion together. Players are able to synchronize and create complex motion without a designated leader or follower. Such synchrony is typically maintained for only a few seconds. Synchrony is then exited, and re-entered. A mathematical model of coupled predictor-corrector equations captures the entry into zero-phase synchrony in the mirror game, by a mechanism in which the two player’s predictors synchronize, in effect agreeing on future motion [59]. However, this model does not account for exit-from-synchrony, because the simulated players remain in zero-phase synchrony forever. Similarly, models such as HKB and the Bingham model [29] remain locked in zero-phase synchrony once it is attained [5,26,65]. It is of interest to generate more realistic models of human joint improvised motion, both in order to understand it better, and in order to provide tools for innovative rehabilitation strategies for patients suffering from social disorders [16,62,64,66,67] and to improve human-computer/robot interactions [62,63,68].

In this study, we analyze experimental data on exit from synchrony in the mirror game and in tracking of computer-provided stimuli, and present a mathematical model for exit-from-synchrony. The model includes a term that accumulates when the players are synchronized, and–when it becomes large- generates a random force that breaks the synchrony. We show that this model captures realistic entry/exit from zero-phase synchrony. The model also captures additional features of human tracking such as damped overshoots when the input signal suddenly stops. The exit-from-synchrony mechanism also provides a diversity to the motions generated in simulated mirror games, which resembles aspects of the complexity of motion generated in human games. We find that the same exit-from-synchrony term can be added to the HKB and Bingham models, allowing them to more realistically describe joint improvised motion.

## Materials and Methods

### Model simulation

Model simulation was performed using the stochastic equation solver of Wolfram Mathematica 10. Model parameter scan was done by a dedicated code in Wolfram Mathematica 10. Co-confident (CC) motion segments detection was done by the Matlab code of [69]. Briefly, CC was defined by considering segments of motion between two zero crossings of velocity. Segments were considered CC if they met two conditions (i) they contained only one acceleration zero crossing (that is, they had no multiple velocity peaks) and (ii) the velocity profiles of the two segments (the stimulus and tracker, or the two mirror game players) were similar in terms of the timing of their zero velocity crossing (time difference <150ms) and had low relative rms velocity difference (<0.95) (see [69] for more details).

### Experimental data

We used data from two previous studies. The tracking data was described in [59,61]. Briefly, 30 subjects held a tablet stylus sampled at 100 Hz and tracked a vertically moving square on a screen with forward-backward arm movements. The stimulus position was a sine wave of piecewise constant period and amplitude, with pieces that lasted 5–40 sec, and frequencies in the range of 0.2–2 Hz.

The mirror game data was described in [60]. The mirror game is a visual interpersonal-coordination task which is open-ended. Briefly, pairs of players moved handles on parallel tracks and their position was sampled at 50Hz, using the device of [59]. Players were instructed to create synchronized and interesting motion. Rounds lasted three minutes, with one player designated as leader and the other follower, or with neither player designated as leader or follower (joint improvisation). Players were able to stay in zero-phase synchrony (and 1–1 frequency) while changing their amplitude and frequency in complex ways even without a designated leader or follower. Videos showed that people are fully attentive and engaged throughout. (Data of representative games is available in supporting information files S1 Dataset through to S13 Dataset).

## Results

### People tracking a piecewise periodic signal show periods of jitter and periods of synchrony

In order to develop the model, we begin by analyzing the way that people track a computer generated signal, using the experimental dataset of [61]. 30 subjects tracked a vertically moving square on a screen with forward-backward arm movements, holding a tablet stylus. The stimulus position was piecewise periodic, made of pieces of sine waves of constant period and amplitude that lasted 5–40 sec. The frequencies in the stimulus were in the range of *f* = 0.2-2Hz.

**Fig 1:**
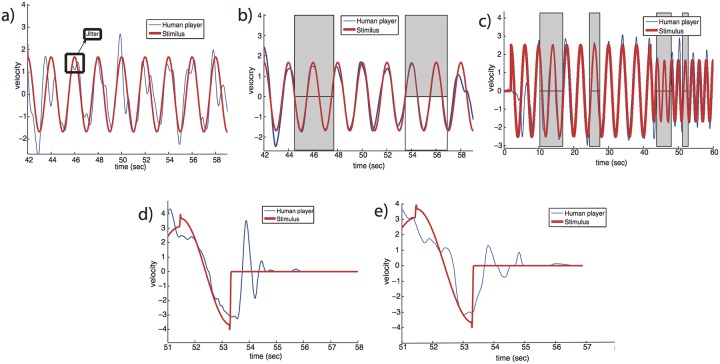
Human tracking (blue) of computer-generated stimuli (red) shows jitter, brief periods of synchrony and transient decay when the stimulus ends. (a) Jittery motion which weaves around the stimulus. In this interval of time, the tracker did not synchronize to the signal according to the criteria described in Methods (CC motion). (b) Velocity trace of a subject which showed two intervals of high zero-phase synchrony with the stimulus (gray boxes), and some jitter when not synchronized. (c) An example of a full round in which the subject entered and exited synchrony with the stimulus. (d, e) When the stimulus suddenly stopped (velocity went to zero discontinuously), subjects showed decaying overshoots and undershoots lasting a few periods. Discontinuities in the stimulus velocity occur at transitions between pieces of constant amplitude and frequency in the stimulus position signal.

We find that over most of the tracking motion, people’s velocity traces weaved around the computer generated input signal, showing undershoots and overshoots of the stimulus velocity. This wavy motion had a mean frequency of about 1 Hz, and is termed *jitter* [70,71] (Fig 1a). This agrees with previous studies of the mirror game, in which followers showed similar jitter around the leaders trajectory [59,61], as well as the earlier work of Miall and colleagues on manual tracking [70–73].

For a fraction of the time, however, players were able to track the stimulus with very little jitter and good precision and zero-phase synchrony (gray boxes in Fig 1b and 1c). These synchronized periods are called co-confident motion (CC), and are detected automatically as defined as in [69]. CC motion in the current dataset occurs in about 5% of the tracking time on average, and for individual people varies between 0% and 11%.

The CC periods lasted 3 sec on average (ranging between 0.5–7.5 sec). After each such synchronized period, the tracker returns to show larger velocity error with the stimulus. We term the phenomenon in which the motion loses zero-phase synchrony “exit-from-synchrony”.

In addition to jitter and exit-from-synchrony, we note an additional feature of human tracking in this dataset, as a benchmark for developing the model in the coming sections. This occurred when the stimulus motion suddenly stopped, and remained at zero velocity. We observe that people show a damped overshoot-undershoot response, settling to zero velocity after 1–3 periods (Fig 1d and 1e). Similar phenomenon occur when people tap their fingers trying to synchronize with an auditory signal, and persist for a tap or two after the signal suddenly stops [74]. This phenomenon suggests that people build an internal expectation of future motion and act by that prediction.

In summary, human tracking of a piecewise periodic computer-generated signal showed jitter except for brief periods of synchrony, exit-from-synchrony, and damped oscillations when stimulus suddenly stopped.

### Predictor-corrector model with friction and exit-from-synchrony (PCFE model) shows similar behavior to human tracking

We compared the tracking data to the predictor corrector (PC) model of Noy et al. 2011. We next describe how the PC model shows disagreement with some of the features observed in the tracking experiment, and then go on to modify the model to capture these features. The PC model is described by Eqs 1–3. It was originally developed to understand entry into synchrony in the mirror game, which is an open-ended joint improvisation task, and is therefore somewhat more complex than minimal models needed to account for tracking or well-defined coordination tasks. To explain behavior in the mirror game, the model required a predictor-corrector design [59]. In the model, the rate of change of velocity of the tracker *v*_1_ is given by a corrector term *f*_1_(*t*) and a predictor based on a sum of periodic functions with time dependent amplitudes *A*_1*n*_(*t*)

$$\frac{dv_{1}}{dt}\;=\;predictor+corrector=\sum_{n}A_{1n}\omega \;n\;cos\left(\omega \;n\;t\right)+f_{1}\left(t\right)$$ (1)

The corrector integrates over the velocity error between the tracker *v*_1_ and the stimulus *v*_2_:

$$\frac{df_{1}}{dt}=k_{1}\;\left(v_{2}-v_{1}\right)$$ (2)

The predictor amplitudes A_1n_ learn the amplitudes of the corresponding frequency components of the stimulus, A_2n_, with rate *g*:

$$\frac{dA_{1n}}{dt}=g\;\left(v_{2}-\sum_{m}A_{1m}\text{sin}\left(\omega \;m\;t\right)\right)\;\text{sin}\left(\omega \;n\;t\right)\;\text{for }n,m=1,2,3\ldots$$ (3)

**Fig 2:**
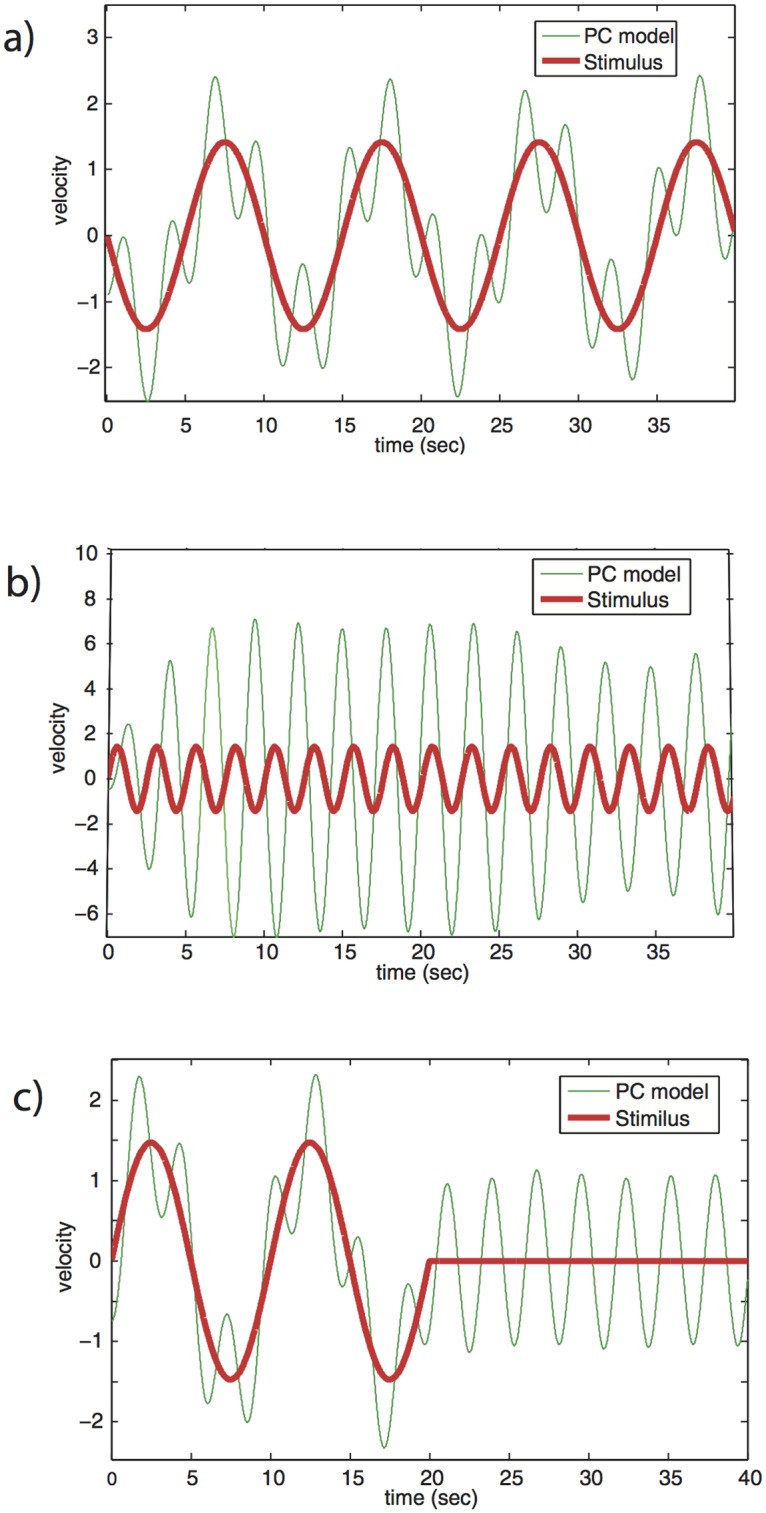
The PC model shows unrealistically high jitter when tracking stimuli. (a) Tracking a stimulus with *f*(0) = 0.1, *A*_21_(0) = −1, initial velocity *v*_1_(0) = −1, *v*_2_(0) = −0.5. Jitter amplitude is about 5-fold higher than observed in Fig 1a. (b) at high stimulus frequency (not obeying $\omega \ll \sqrt{k}$), *f*(0) = 2, *A*_21_(0) = 1.5, *v*_1_(0) = 0.5, *v*_2_(0) = −0.5, jitter increases to an amplitude to be almost 4 times higher than the stimulus and 20-fold higher than observed in Fig 1a. Such phenomena occur for a wide range of parameters. Here we used *k* = 8, *g* = 0.3 and initial amplitudes *A*_1*n*_(0) = 0. (c) When stimulus suddenly stops, PC model does not settle to zero velocity. Here we use three periods in the predictor, and *f*(0) = 0.1, *A*_21_(0) = −1, *v*_1_(0) = 0.5, *v*_2_(0) = −0.5 and all other initial amplitudes *A*_*in*_(0) = 0.

We find that the PC model shows qualitative and quantitative disagreement with the measured tracking behavior, similar to the recent findings of [62]. The PC model shows jitter amplitude that is an order of magnitude larger than observed (compare Fig 2a and 2b vs. Fig 1a, 1b and 1c). The jitter amplitude in the model is sensitively dependent on the initial conditions. When stimulus velocity goes to zero, the model motion continues to jitter indefinitely (Fig 2c), as opposed to the observed decay to zero velocity by human trackers (Fig 1d and 1e).

In light of these disagreements, and specifically in order to resolve the over-jittery pattern, we added a damping term –*αf* to the corrector Eq (2):

$$\frac{df_{1}}{dt}=k\left(v_{2}-v_{1}\right)-\alpha \;f_{1}$$ (4)

The corrector equation is the source of jitter: jitter frequency is given by the corrector constant *k*_1_ (jitter frequency equals $\sqrt{k_{1}}$), which effectively acts as a spring that connects *v*_1_ and *v*_2_. The damping term causes the jitter amplitude to decrease. The simulated player becomes synchronized to the stimulus after a time scale of $\frac{1}{\alpha }$, because jitter is eliminated by the friction term. Thereafter, the model remains in synchrony forever. Hence, adding the damping term alone solves the high jitter problem, but leads to unrealistic over-synchrony. We thus sought a new mechanism for exit-from-synchrony.

In order to model exit-from-synchrony we first attempted a simple approach: adding a random disturbance to the velocity [75]. Adding such random noise continuously to the equations did not allow them to reach synchronization. Thus, we sought a way to add noise only when the players have been synchronized for some time. To achieve this, we need to measure the error between the players, suitably integrated over the recent past, which we denote E. We then control the noise amplitude according to a function Q(E) that monotonically decreases to zero as function of E.

$$\frac{df_{1}}{dt}=k\left(v_{2}-v_{1}\right)-\alpha \;f_{1}+\text{Q}\left(\text{E}\right)\;$$ (5)

We tested various forms of Q(E), and chose a form that is both simple, and adequately captures the experimental results. This model, described in Eqs 6–9, is termed the predictor-corrector-with-friction-and-exit-from-synchrony model (PCFE).

The velocity and amplitude equations are unchanged and contain the predictor and corrector terms described above:

$$\;\frac{dv_{1}}{dt}=\sum_{n}A_{1n}\omega \;n\;cos\left(\omega \;n\;t\right)+f_{1}$$ (6)

$$\frac{dA_{1n}}{dt}=g\;\left(v_{2}-\sum_{m}A_{1m}\text{sin}\left(\omega \;m\;t\right)\right)\;\text{sin}\left(\omega \;n\;t\right)$$ (7)

The corrector *f*_1_ accumulates the differences in velocity as in the PC model, has a friction term with coefficient *α*. It also has a new term, an exit-from-synchrony term with strength *β*_1_, proportional to a white noise term *ξ*_1_ (new terms are in bold)

$$\frac{df_{1}}{dt}=k\left(v_{2}-v_{1}\right)-\alpha \;f_{1}+\beta_{1}\frac{v_{2}}{1+\frac{E}{E_{0}}}\xi_{1}$$ (8)

The white noise amplitude, $\beta_{1}\frac{v_{2}}{1+\frac{E}{E_{0}}}$, in the exit-from-synchrony term decreases with increasing values of the error *E* (described below) between the stimulus and the trackers velocity, when E is comparable or smaller than a threshold error E_0_. Furthermore, the noise amplitude is proportional to *v*_2_. This is needed so that when the input signal *v*_2_ stops and stays at zero velocity, the exit-from-synchrony term also goes to zero: otherwise the simulated player,v_1_, would converge to zero velocity, which will cause E to decrease and later cause v_1_ to show random noise around zero (whereas human players stay at zero velocity without fluctuations, Fig 1d and 1e). In summary, the exit-from-synchrony term randomly kicks the velocity in times of synchrony, except when synchronizing to zero velocity.

To define the error E in the exit-from-synchrony equation, we use the relative RMS error of the velocities *v*_1_ and *v*_2_, integrated over time τ. To define E, we compute the squared differences of velocities D and squared sum of velocities S

$$\frac{dD}{dt}={\left(v_{1}-v_{2}\right)}^{2}-\frac{D}{\tau }$$ (9)

$$\frac{dS}{dt}={\left(v_{1}+v_{2}\right)}^{2}-\frac{S}{\tau }$$ (10)

and take their ratio

$$E=\frac{D}{S+\epsilon }$$ (11)

The small parameter *ϵ* (*e*. *g*., *ϵ* = 0.001) is introduced in order to avoid divergence when S is zero. The parameter *ϵ* has negligible effect on the model, except for a transient at the beginning of motion.

It is also possible to add the noise factor, $\beta_{1}\frac{v_{2}}{1+\frac{E}{E_{0}}}\xi_{1},$ directly in to the velocity Eq 6 –but the discontinuity of the noise, *ξ*_1_, causes *v*_1_ to be not as smooth as the observed motion. Adding the noise term to the corrector Eq (8) effectively integrates over the noise, causing the trajectory of *v*_1_ to be smoother and still maintain its ability to exit-from-synchrony.

### Calibrating the model

The PCFE model has several parameters. It shares with the original PC model the corrector strength *k* and the predictor rate *g*. The corrector strength *k* determines the jitter frequency [59]: $\omega_{jitter}=\sqrt{k}$, the frequency at which the follower overshoots and undershoots the leaders trajectory. Since our experimental data shows jitter with average frequency of about 1Hz [59,61], we set $k=\omega_{jitter}^{2}=10\;{\text{sec}}^{-2}$ (note the 2*π* conversion factor between frequency and angular frequency).

The PCFE model also has four new parameters: the friction coefficient *α*, and the exit-from-synchrony amplitude *β*, the error threshold E_0_ and the time over which error is integrated *τ*. We determined model parameters to come as close as possible to the observed RMS error of the tracker and stimulus in three experimental rounds. We find a set of parameters that shows good agreement with the observed RMS errors averaged over all players. The parameters are = 0.3 sec^−1^, *α* = 4 sec^−1^, *β* = 6, E_0_ = 0.05 and *τ* = 2 sec. These values of E_0_ and *τ*means that exit-from-synchrony occurs when the integrated relative error is lower than about $\sqrt{{\text{E}}_{0}}≅0.2$ for about *τ* = 2 seconds. Sensitivity analysis shows that the model fit to the data is robust to sizable variations in the parameters (~two-fold in *k*, *g*, E_0_ and *τ*. and ~20% in *α* and *β*).

**Fig 3:**
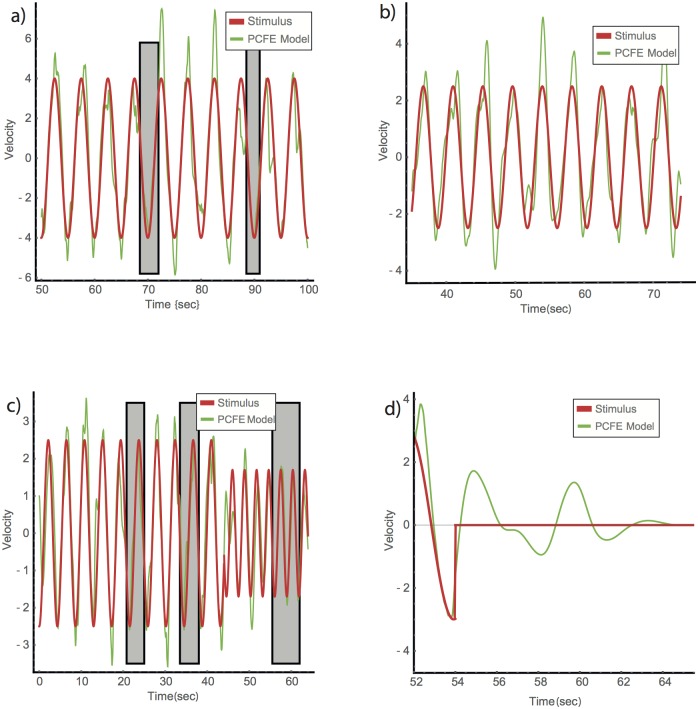
Model with friction and exit-from-synchrony (PCFE) tracks an input signal with realistic jitter and shows entry and exit from synchrony. (a) The PCFE model shows dynamics qualitatively similar to human players, with the best fit parameter set. (b) PCFE model with parameters that produce high-error tracking with jitter similar to the human player in Fig 1a. Parameters are same as in (a) except for a lower corrector damping parameter, *α* = 1. (c) High precision tracking to a changing stimulus with frequent entry and exit-from-synchrony, similar to the subjects in Fig 1b, is obtained with PCFE model with *α* = 5. (d) When stimulus suddenly stops, PCFE model converges to zero velocity after a few periods, as in Fig 1d. Model parameters: *k* = 8, *g* = 0.3, *τ* = 1, *E*_0_ = 0.04, *β* = 3, *α* = 4, *v*_1_(0) = 1, *v*_2_(0) = −1, *A*_11_(0) = 0.2, and *A*_12_(0) = *A*_13_(0) = 0, except as noted.

The PCFE model with these parameters reproduces human-like tracking behavior in our experimental dataset (Fig 3a). It shows appropriate jitter amplitude, enters synchrony for periods of about 1–4 sec and has a prevalence of high-synchrony (CC) events similar to that observed in human Subjects (5%).

By varying the parameters, the model can also simulate the variation in tracking behavior of different subjects following the same stimuli. For example, relatively high-error tracking (Fig 3b) similar to the subject motion shown in Fig 1a, can be achieved by decreasing *α* from 4 to 1 (while keeping all other parameters the same). Simulating a high-synchrony player which exits and re-enters synchrony, similar to the round shown in Fig 1b, is achieved by increasing the friction parameter, *α* from 4 to 5 (Fig 3c). The model also shows convergence to zero velocity upon stimulus termination, within about 2.5 periods (Fig 3d- compare to Fig 1d and 1e). The number of periods is most sensitive to the parameters g and *α*. We conclude that the PCFE model describes aspects of human tracking in the present context reasonably well.

### Mirrored PCFE model accounts for aspects of human dyad behavior in the mirror game

**Fig 4:**
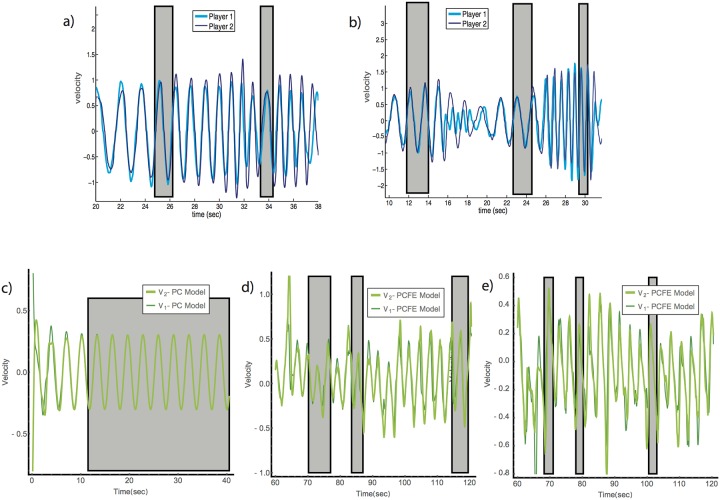
Mirrored PCFE models capture key aspects of human joint improvised motion in the mirror game. (a, b) Examples of JI rounds by human players from the data of Ref. [59]. Periods of co-confident motion (CC) are in gray boxes. (c) PC model simulation converges to synchrony and does not exit it. Here *f*_1_(0) = *f*_2_(0) = 0, *k*_1_ = *k*_2_ = 8, *g*_1_ = *g*_2_ = 0.5, *v*_1_(0) = 1 *v*_2_(0) = −1, *A*_11_(0) = 0.2, and all other initial amplitudes *A*_*ji*_(0) = 0. (d, e) Mirrored PCFE model dynamics show more complexity and realistic entry and exit from synchrony. Parameters of both mirrored PCFE equations are identical and equal to the best fit parameters found in the tracking experiment, except for (e) where *β* = 1 is used to describe a player dyad with lower exit-from-synchrony amplitude.

We next asked whether the PCFE model can also capture joint human improvisation in the mirror game. The mirror game includes two types of rounds: leader-follower and joint-improvisation. In leader-follower rounds one player leads and the other follows, in essence a tracking of a human leader. In joint-improvisation rounds (JI) there is no designated leader and follower. Players show jittery motion, and, for a fraction of the time, are able to enter into a state of complex motion with no jitter and high zero-phase synchrony (Fig 4a and 4b), namely CC motion.

As shown in Ref [59], the original PC model is able to exhibit entry into CC, where the JI condition is modeled by two PC modules in a mirror configuration, such that the output of one is fed as the stimulus input to the other. However, unlike human players, the PC stays locked in the CC state because it has no exit-from-synchrony mechanism (Fig 4c, shaded gray area shows CC region).

To simulate the JI condition, we joined two PCFE model controllers in a mirror configuration. One player is represented by Eqs 6–11, and the second player by the same equations, with *v*_1_ and *v*_2_ switched. We used identical parameters for the two players- equal to the parameters set calibrated from the tracking experiments described above. We find that the model is able to enter CC-like motion. However, unlike the PC model which stays locked in CC motion, the PCFE model leaves zero-phase synchrony after a few seconds (as determined by the parameters *τ* and E_0_), and then re-enters zero-phase synchrony, and so on (Fig 4d and 4e). This mimics human behavior in the mirror game (compare human motion in Fig 4a and 4b, to model motion in Fig 4d and 4e).

Remarkably, although the parameters were calibrated from tracking experiments, the mirrored model showed both quantitative and qualitative agreement with the mirror-game experiment. The model shows a fraction of time in CC, 8.5±3%, that is close to that of human players, e.g. 9±2% observed in Ref [60]. The time to the first CC event in the model averages 38±4 sec, which is similar to the human average of 41±6 sec.

The exit-from-synchrony term in the equation adds a random component to the simulated motion, producing diversity in the motion that is not found in the PC model. The motion does not settle into periodic behavior, but instead varies between high and low amplitudes, and different frequencies. The higher the random force amplitude *β*, and the larger the predictor learning rate *g*, the more pronounced the complexity and diversity of the motion generated by this randomization effect. This is in contrast to purely deterministic models which tend to converge to periodic motion. Thus, exit-from-synchrony may be a mechanism to explain part of the complexity of motion generated by human players in the mirror game.

### Exit-from-synchrony in the HKB and Bingham models

We tested the generality of the exit-from-synchrony mechanism, by applying it to additional models of human synchronization. We begin with the well-studied HKB model [26,62,63]. The HKB model includes a nonlinear oscillator term for each simulated person. It combines a Van-der-Pol and a Rayleigh oscillator (parameters *ϵ*, *ω*_1_, *γ*), and a nonlinear coupling term which acts to make the two person velocities equal (parameters *α*, *β*).

$$\frac{{d\text{v}}_{\text{i}}}{dt}=-\epsilon \;{\text{v}}_{\text{i}}-\omega_{\text{i}}{}^{2}{\text{x}}_{\text{i}}-\gamma {\text{x}}_{\text{i}}^{2}{\text{v}}_{\text{i}}-\delta {\text{v}}_{\text{i}}^{3}-\left({\text{v}}_{\text{i}}-{\text{v}}_{\text{j}}\right)\left(\alpha +\beta {\left({\text{x}}_{\text{i}}-{\text{x}}_{\text{j}}\right)}^{2}\right),$$ (12)

**Fig 5:**
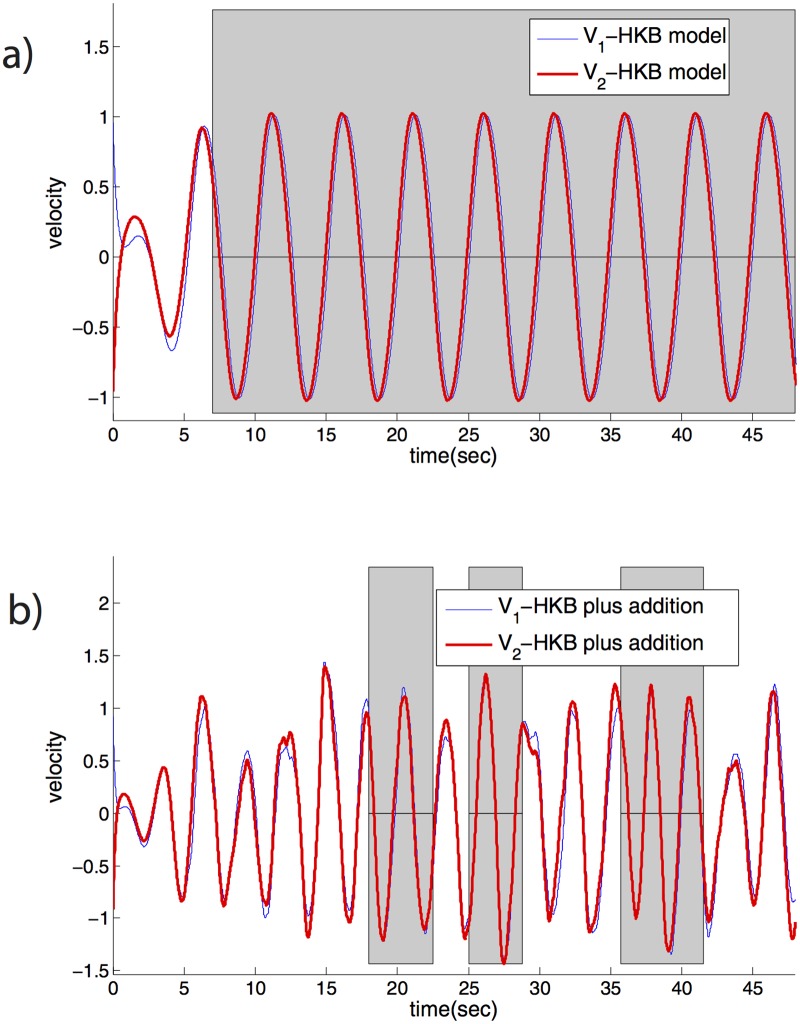
HKB model with an added exit-from-synchrony term shows entry and exit-from-synchrony. (a) Original HKB model of Ref [26] converges to synchrony and stays locked in a periodic synchronized motion. *ω*_1_ = 1.5, *ω*_2_ = 1, *ϵ* = *−*1, *β* = 0.5, *δ* = 1, *α* = 4, *γ* = 1, *v*_1_(0) = 2, *v*_2_(0) = 1, *x*_1_(0) = *x*_2_(0) = 0. (b) Modified HKB model according to Eqs 13 and 14, with exit-from-synchrony term, shows entry and exit-from-synchrony and complex non-periodic motion. Parameters are the same as in a), with exit-from-synchrony parameters *β*_1_ = *β*_2_ = 3, *τ* = 1, *E*_*0*_ = 0.04, *ϵ*_0_ = 0.001, *η* = 2.

The dynamics of the model converge to synchronized motion in phase or antiphase. Once synchrony is reached, the motion remains locked in a periodic trajectory forever (Fig 5a).

We added an exit-from-synchrony term, the same term as in the PCFE model. The new model is described by the following equations (new terms are marked in bold).

$$\;\;\frac{{d\text{v}}_{\text{i}}}{dt}=-\epsilon \;{\text{v}}_{\text{i}}-\omega_{\text{i}}{}^{2}{\text{x}}_{\text{i}}-\gamma {\text{x}}_{\text{i}}^{2}{\text{v}}_{\text{i}}-\delta {\text{v}}_{\text{i}}^{3}-\left({\text{v}}_{\text{i}}-{\text{v}}_{\text{j}}\right)\left(\alpha +\beta {\left({\text{x}}_{\text{i}}-{\text{x}}_{\text{j}}\right)}^{2}\right)+f_{i}\left[t\right]$$ (13)

$$\frac{df_{i}}{dt}=-\eta \;f_{i}+\beta_{i}\frac{v_{j}}{1+\frac{E}{E_{0}}}\xi_{i}$$ (14)

The error E is calculated as in Eqs 9–11. Simulating this model shows that the players enter zero-phase synchrony, exit zero-phase synchrony and then re-enter, in a way reminiscent of human players. The motion, as in PCFE, has a complexity and diversity introduced by the random nature of the exit-from-synchrony term (Fig 5b).

We further tested the HKB model in tracking mode (uni-directional coupling), in which the velocity v2 is a given sinusoidal wave. We find that the exit-from-synchrony term allows the HKB model to avoid remaining locked in zero-phase synchrony, and instead to show realistic exit-and-entry into synchrony also in tracking mode (see Figure A in S1 File).

Finally, we tested the Bingham model of coupled motion [29,30]. The advance provided by the Bingham model was to include perception explicitly when modelling rhythmic coordination. It models each player as a damped linear oscillator:

$$\ddot{x_{1}}+b\dot{x_{1}}+k\;x_{1}=c\;sin\left(\phi_{2}\right)P_{12}$$ (15)

$$\ddot{x_{2}}+b\dot{x_{2}}+k\;x_{2}=c\;sin\left(\phi_{1}\right)P_{21}$$ (16)

With a nonlinear coupling that includes a noise term *N*_*t*_

$$P_{12}=sgn\left[sin\left(\phi_{1}\right)*sin\left(\phi_{2}\right)+\alpha {\left(\dot{x_{1}}-\dot{x_{2}}\right)}^{3}N_{t}\right]$$ (17)

**Fig 6:**
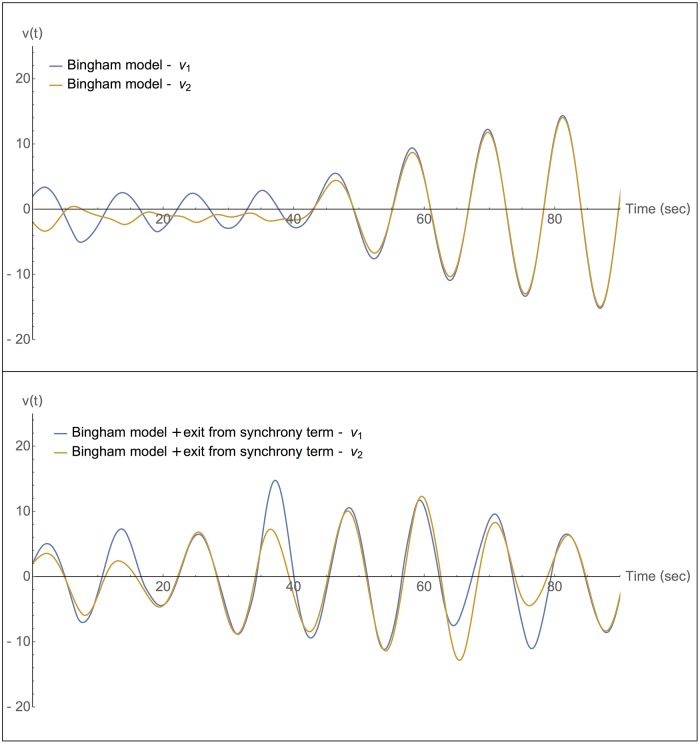
Bingham model with an added exit-from-synchrony term shows entry and exit-from-synchrony. (a) Original Bingham model of Ref [29,30] with out-of-phase initial conditions converges to synchrony and stays locked in a periodic synchronized motion. Parameters: *c* = 0.7, *b* = 0.35, *k* = 0.3, *α* = 0.3, x10 = 2, x20 = −2, v10 = 1, v20 = −1, (b) Modified Bingham model with exit-from-synchrony term shows entry and exit-from-synchrony and complex non-periodic motion. Parameters are *c* = 0.7, *b* = 0.35, *k* = 0.3, *α* = 0.3, x10 = 2, x20 = 2, v10 = 2, v20 = 1, β = 3, τ = 12, E0 = 0.001, ϵ = 0.001.

Note that the noise amplitude in the equation above is proportional to an asymmetric function of the difference between the player velocities, ${\left(\dot{x_{1}}-\dot{x_{2}}\right)}^{3}$. Thus, when the two players are synchronized in anti-phase, the noise term is large and can cause anti-phase synchrony to become unstable; the dynamics then converge to zero-phase synchrony (Fig 6a). At zero-phase synchrony, in contrast, the noise term has zero amplitude (since $\dot{x_{1}}=\dot{x_{2}}$). In addition, in that state the *sin*(ø_1_) * sin(ø_2_) term is positive and as a result P_ij_ is one. Therefore, once entered, zero-phase synchrony is stable and the model remains locked in zero-phase synchrony (Fig 6a).

We next added the exit-from-synchrony term introduced in the present study to the Bingham model, by changing the equation for the coupling term Pij:

$$P_{ij}=sgn\left[sin\left(\phi_{1}\right)*sin\left(\phi_{2}\right)+\alpha {\left(\dot{x_{1}}-\dot{x_{2}}\right)}^{3}N_{t}\;\right]\;+\beta \frac{\dot{x_{j}}}{1+\frac{E}{E_{0}}}\xi$$ (18)

The error *E* is calculated as described in Eqs 9–11. In this context it is apparent that the exit-from-synchrony term takes into account perceptual information. We find that this extended model shows more realistic exit-and-entry into zero-phase synchrony (Fig 6b).

## Discussion

This study focused on the phenomenon of exit-from-synchrony. We provide evidence of exit-from-synchrony in joint improvisation in the mirror game, and when people track a computer-generated signal. People enter periods of zero-phase synchrony with each other or with the computer-generated signal, but exit this synchrony after a few seconds, and later re-enter synchrony and so on. Most previous mathematical models of human coordination account for entry into synchrony, but exit from zero-phase synchrony is more rarely studied [30][57]. We presented a model that captures this important feature of human tracking and mirror game motion. The model includes an exit-from-synchrony term: the motion receives random kicks after periods of low velocity error between players. The model also quantitatively captures the overshoot people exhibit when the signal suddenly stops. An exit-from-synchrony term seems to be general since it can be added also to the HKB and Bingham models for human coordination. It adds realistic complexity to simulated motion by allowing escape form a purely periodic solution. This study thus is step towards understanding how, in tasks that require motion coordination, people exit and re-enter periods of high zero-phase synchrony.

Exiting synchrony may allow for discovery of new possibilities in the interaction. Thus it may represent a form of novelty seeking [76]. For example, in the mirror game model, exit-from-synchrony introduces a perturbation which generates a more complex motion than the periodic limit cycle reached without the exit-from-synchrony term. The present study attempts to experimentally characterize and mathematically model this aspect of human interaction in a simple motion paradigm. We hope it can form the basis of a more complete exploration in complex interaction settings.

Our experience in the mirror game, and more generally in theatre improvisation, suggests that there may be additional subtlety to the way people exit from synchrony that is not captured by the present mathematical models. In the mirror game, we observe that players break synchrony in a number of ways. One way is going to extremes- in the mirror game this is seen when players go to high frequency (above 1 Hz) and lose zero-phase synchrony [30]. Another way is to suddenly stop. A third is to turn on a completely different motion program- to suddenly change amplitude, period, or nature of the motion. The present mathematical model adds a random force to break synchrony; future work can add diverse exit-from-synchrony mechanisms to the model, for example by setting completely new amplitudes for the predictor *A*_*i*_(*t*).

We find that people show different tracking and mirror-game styles [60], with systematically different levels of jitter and CC motion. Such variation can be modelled using the present framework: for example, jitter amplitude of different players can be modeled by varying the corrector damping rate alpha. Future experimental and theoretical work can further explore in more detail the individuality of human players in the ways that they enter and exit from synchrony.

The present approach may be used for designing realistic computer simulated motion in treatment and rehabilitation settings that employ the mirror game, complementing the work of [62,63]. Extensions of this approach might be useful for generating co-creative and non-boring robot-human or avatar-human interactions [58], in which the robot or avatar is appropriately affected by the non-verbal communication of the human partner. In contexts where productive human-computer interfaces are needed, exit-from-synchrony may provide ways to make the interaction more engaging, exploratory and allow repair of synchrony ruptures that can enhance relationship.

## Supporting Information

S1 File Adding the exit-from-synchrony term to a unidirectional coupling HKB model provides it with entry/exit dynamics from zero-phase tracking. (DOCX)

S1 Dataset Position of 30 human players reacting to the stimulus 1. (MAT)

S2 Dataset Position of 30 human players reacting to the stimulus 2. (MAT)

S3 Dataset Position of 30 human players reacting to the stimulus 3. (MAT)

S4 Dataset Position of 30 human players reacting to the stimulus 4. (MAT)

S5 Dataset Position of 30 human players reacting to the stimulus 5. (MAT)

S6 Dataset Position of 30 human players reacting to the stimulus 6. (MAT)

S7 Dataset Position of 30 human players reacting to the stimulus 7. (MAT)

S8 Dataset Position of 30 human players reacting to the stimulus 8. (MAT)

S9 Dataset Position of 30 human players reacting to the stimulus 9. (MAT)

S10 Dataset Position of 30 human players reacting to the stimulus 10. (MAT)

S11 Dataset Position of 30 human players reacting to the stimulus 11. (MAT)

S12 Dataset Stimuli used in the experiments. Piecewise periodic sin waves. (MAT)

S13 Dataset Mirror game data. Rounds between human playing each other. (MAT)

## Abbreviations

CC: Co-Confident motion
HKB: Haken, Kelso and Bunz model
JI: Joint Improvisation
LF: Leader Follower
PC: Predictor Corrector
PCFE: Predictor Corrector with Friction and Exit-from-synchrony
