# Supplementary material for: Exit from Synchrony in Joint Improvised Motion
Source: PLoS One. 2016 Oct 6;11(10):e0160747. doi: 10.1371/journal.pone.0160747 (PMC5053605; doi:10.1371/journal.pone.0160747)
Supplement: S1 File — (DOCX) [file pone.0160747.s001.docx]

**Exit from synchrony in joint improvised motion - Supplementary Information**

Assi Dahan^1,2^, Lior Noy^1,2^, Yuval Hart^1,2^, Avi Mayo^1,2^, Uri Alon^1,2*^

1 Department of Molecular Cell Biology,­­­­­ Weizmann Institute of Science, Rehovot, Israel
 2 Theatre Lab, Weizmann Institute of Science, Rehovot, Israel

**Contents:**

1. **Adding the exit-from-synchrony term to a unidirectional coupling HKB model provides it with entry/exit dynamics from zero-phase tracking…………………………..................................................................................2**
2. **Data set explanations………………………….............................................................3**

**1. Adding the exit-from-synchrony term to a unidirectional coupling HKB model provides it with entry/exit dynamics from zero-phase tracking**

The HKB model can be simulated in unidirectional coupling mode, which is a model for tracking of a given input signal. Such a model, tracking a sine-wave input, locks into zero-phase synchrony and does not show exit form synchrony. We therefore tested a unidirectional-coupling version of the HKB model, to which we added the exit form synchrony term. The model is identical to Eq. 12-16 in the main text for x1 and v1, treating x2(t) and v2(t) as given input signals.

We find that this exit-from-synchrony unidirectional-coupling HKB model shows exit-and-entry from zero-phase synchrony (Fig. S1) that qualitatively resembles the human tracking data (compare to Fig. 1).

­- HKB **+** exit-from-synchrony term

- Stimulus

V(t)

**Figure A. Adding the exit-form-synchrony term to the HKB model in tracking mode (uni-directional coupling) shows exit and entry into zero-phase synchrony.**

**Model param­eters:**

$\omega_{1}=1.5, \omega_{2}=1, \epsilon=-1, \beta=0.5, \delta=1, \alpha=4 , \gamma=1 , v_{1}\left( 0 \right)=2, v_{2}\left( 0 \right)=1, x_{1}\left( 0 \right)= x_{2}(0)=0$.

1. **Data set explanations**

The following datasets are from experiments with the tracking mode-game setup:

S.mat - position of the stimuli used in the experiments- we used rounds 9,10,11. Those rounds were made of a piecewise periodic sin waves.

R1.mat to R11.mat contains the position of 30 human players reacting to the stimulus S. In this study we used round 9,10,11. We converted their location to velocity by taking a numerical derivative.

The Mirror game data.mat are from experiments with the 1D mirror-game setup

They are the recordings of rounds between human playing each other. We used the Joint improvisation data in this paper.
